# Supplementary material for: Convergent evolution links molybdenum insertase domains with organism-specific sequences
Source: Commun Biol. 2024 Oct 18;7:1352. doi: 10.1038/s42003-024-07073-w (PMC11489736; doi:10.1038/s42003-024-07073-w)
Supplement: Supplementary file 1 — Supplementary Information [file 42003_2024_7073_MOESM1_ESM.pdf]

Supplementary Figures

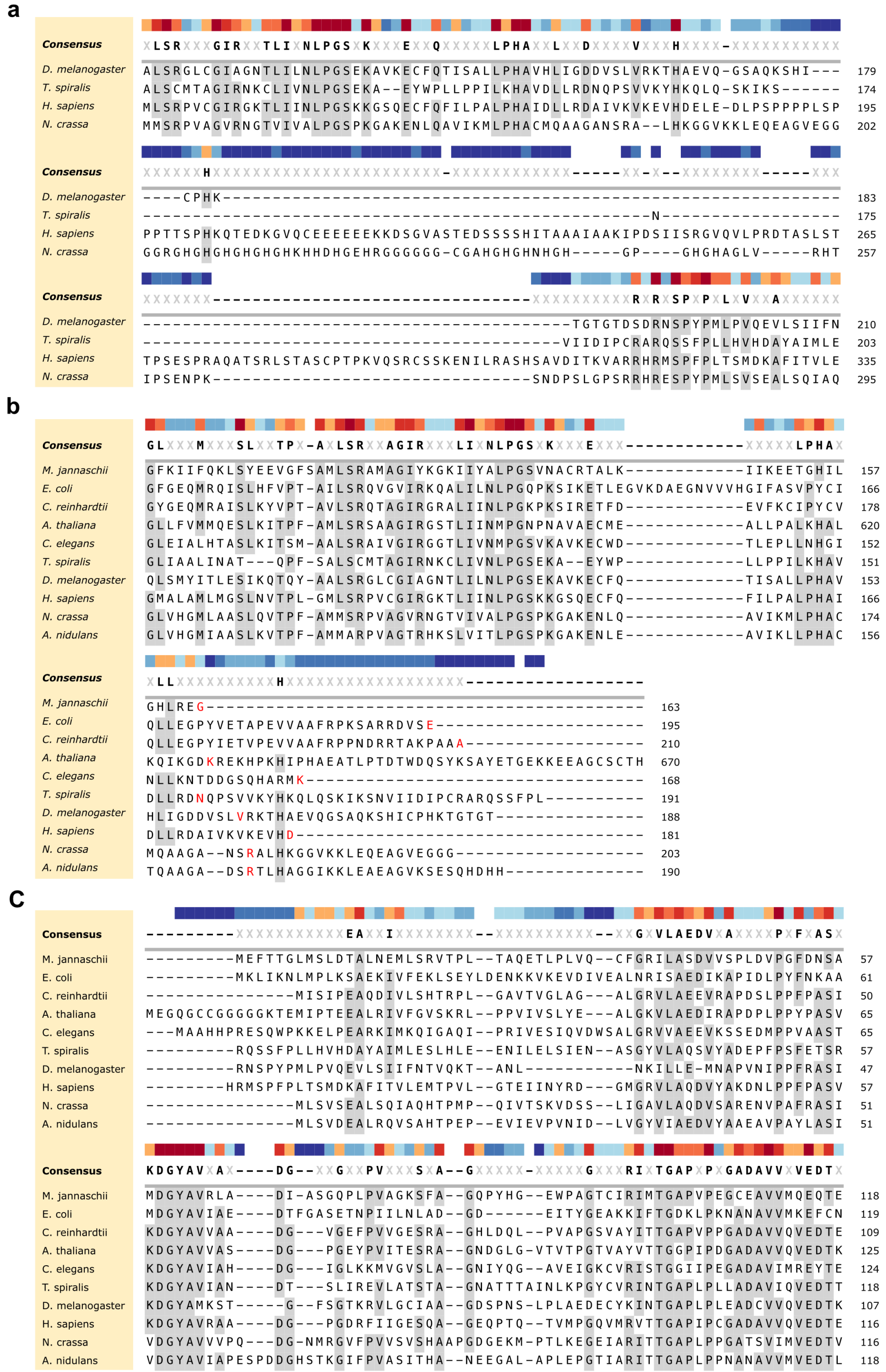

Supplementary Figure 1 | Comparative analysis of Mo insertase domain boundaries. a Alignment of full-length Mo insertase protein with both domains, the linkage region across animal and fungal Mo insertases are shown. **b** Alignment of the G domain proteins, C-terminal region is shown. The domain borders of the estimated G domain are highlighted in red. **c** Alignment of E domain proteins, N-terminal region bordering the E domain is shown. Multiple sequence alignment was performed using MAFFT. Sequence conservation is depicted using a color gradient from blue (lowest) to red (highest), with gray highlights indicating conserved residues.

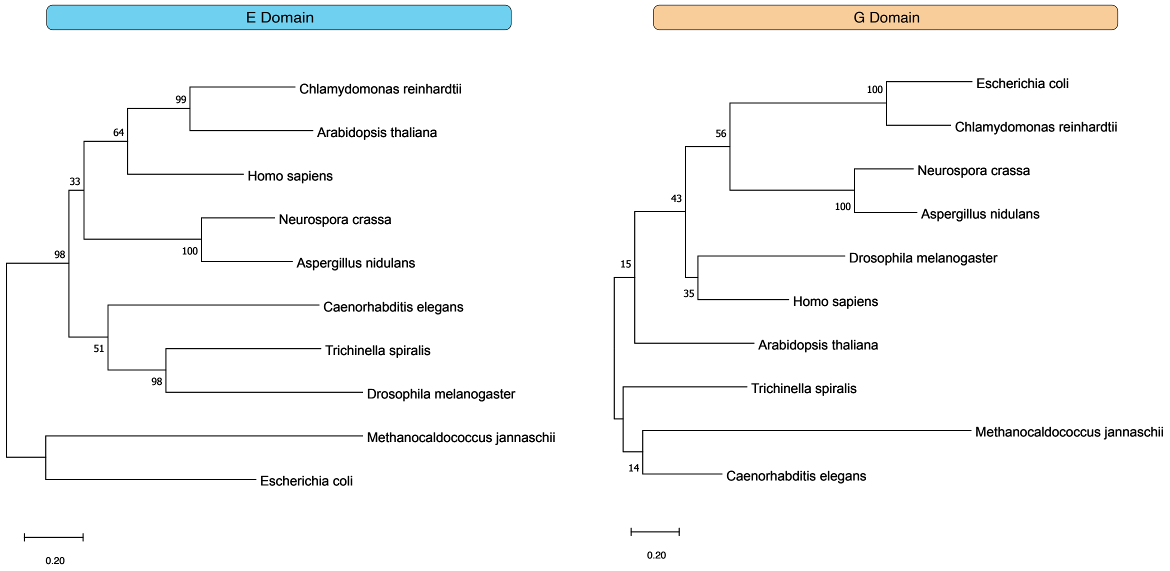

Supplementary Figure 2 | Phylogenetic analysis of Mo insertase domains by Maximum Likelihood method. The evolutionary history was inferred by using the Maximum Likelihood method and JTT matrix-based model. Both trees with the highest log likelihood (-9455.98 E domain, -4001.28 G domain) are shown. The percentage of trees in which the associated taxa clustered together is shown next to the branches. Initial trees for the heuristic search were obtained automatically by applying Neighbor-Join and BioNJ algorithms to a matrix of pairwise distances estimated using the JTT model, and then selecting the topology with superior log likelihood value. The tree is drawn to scale, with branch lengths measured in the number of substitutions per site. This analysis involved 10 amino acid sequences. There were a total of 549 positions in the final dataset. Evolutionary analyses were conducted in MEGA11.

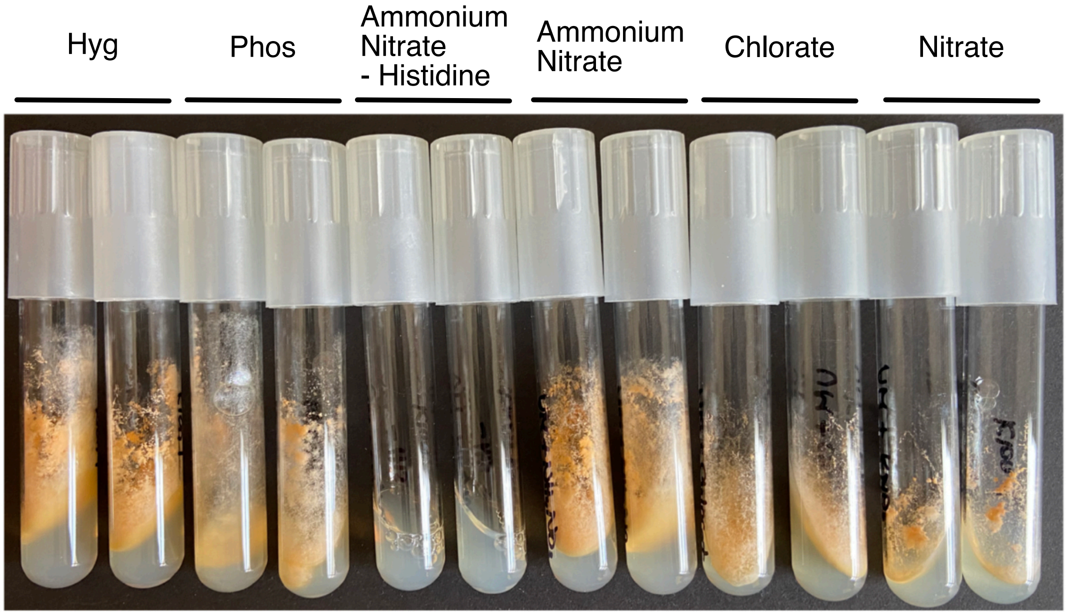

Supplementary Figure 3 | Genotyping of *Δ nit-9*; *his-3*$^{-}$ strain under various growth conditions. The *Δ nit-9*; *his-3*$^{-}$ strain was generated by crossing *his-3*$^{-}$ *mus52::bar mat A*(FGSC988) with *NCU09746::hph mus52::bar mat a* (FGSC18574), resulting in a novel strain harboring both a *his-3* cassette for subsequent transformation and a knock-out in the *nit-9* gene. Verification was performed using different growth media, including hygromycin (hyg), phosphinothricin (Phos), ammonium nitrate, chlorate, and nitrate media, with or without histidine supplementation.

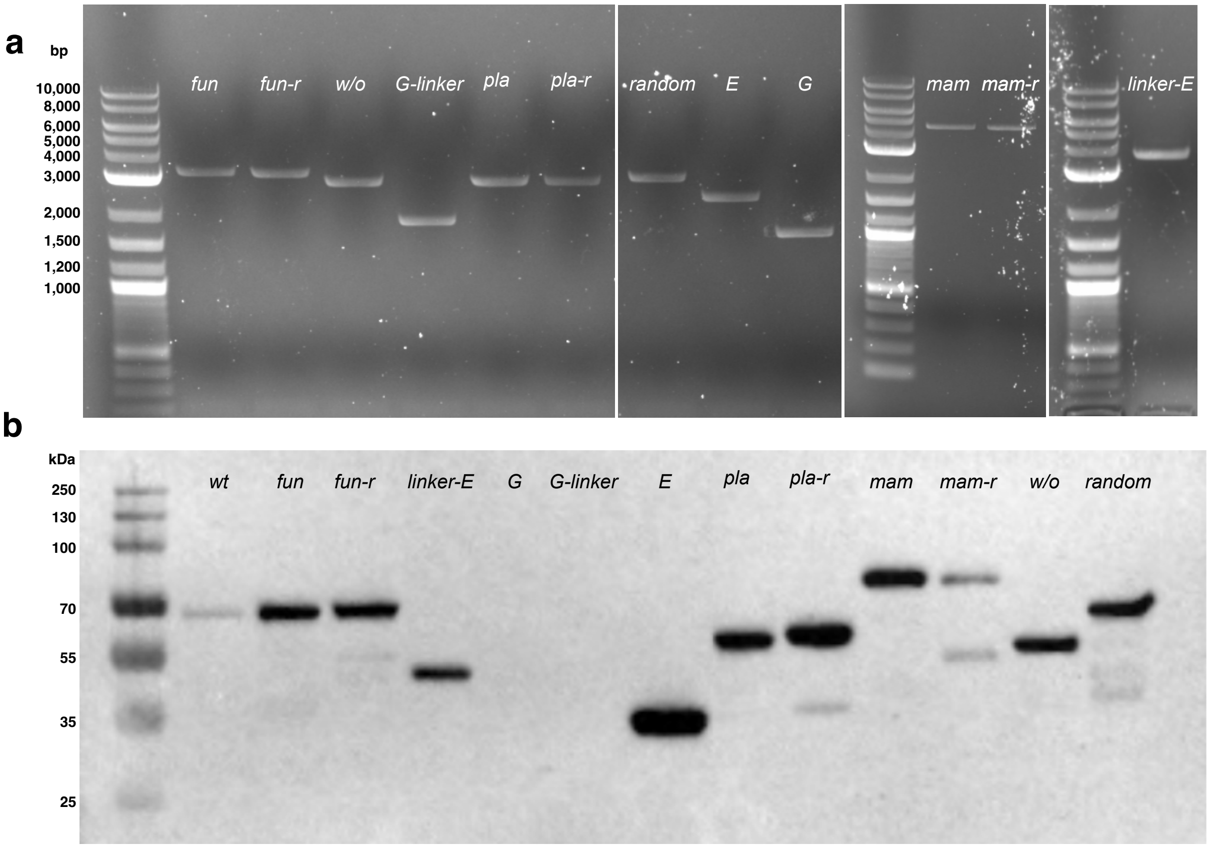

Supplementary Figure 4 | Validation of NIT-9 construct integration at the *his-3* locus. a Gel electrophoresis (1% agarose) was used to confirm transformants by PCR and verify the target gene. Subsequently, the bands were excised and sequenced via Sanger sequencing. **b** Western Blot to confirm protein expression of the expressed constructs. Each lane was loaded with 50 µg of *N. crassa* crude extract. The wild-type (*wt*) and *nit-9 N. crassa* strains are shown. Detection of the NIT-9E domain was achieved using a specific α-NIT-9E antibody. Size was determined using PageRuler Plus Prestained (Cat. #26620 Thermo Scientific Waltham, USA). Abbreviations: fun = fungal, fun-r = fungal reverse, w/o = without linkage region, linker-E, G-linker, pla = plant, pla-r = plant reverse, mam = mammalian. mam-r = mammalian reverse, random, E = E domain, G = G domain.

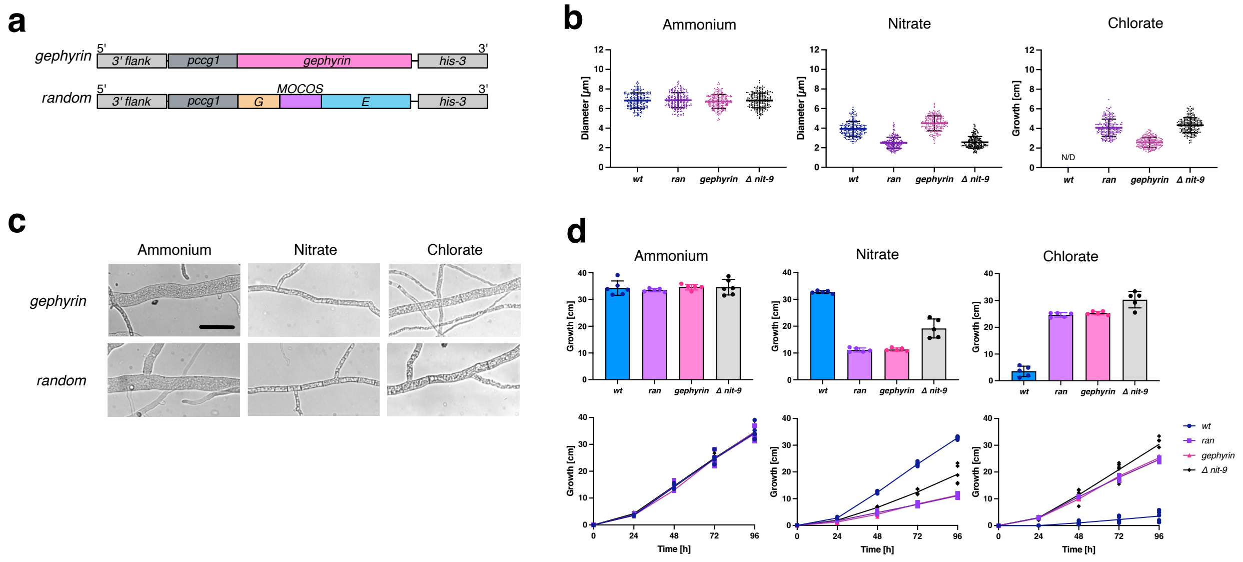

Supplementary Figure 5 | Gephyrin and random sequence linkage fails to recover *Δ nit-9*. a Abbreviated schematic of NIT-9 variants expressed by the *ccg1* promoter in the *his-3* locus of an *N. crassa nit-9* knock-out strain. Strain names: *random* = NIT-9 linked by the 81 amino acid linkage sequence of MOCOS (Q96EN8) from *H. sapiens*. *gephyrin* = Gephyrin isoform X2 (Q9NQX3) from *H. sapiens*. **b** Diameter of *N. crassa* main hyphae was measured after 24 h of growth on Vogel's medium (MM) agar plates (n = 200, mean ± SD, *P <0.001). **c** Hyphae of *N. crassa*, analyzed using bright-field microscopy, bar indicates 10 µm. **d** Race tubes were grown at 30°C in the darkness; the tubes were marked every 24 h. Growth was measured over 96 h. Experiments were performed in six independent replicates (n = 5–6, mean ± SD, *P <0.01, One-way ANOVA). (B-D) All fungi were cultivated with either 80 mM ammonium or 80 mM nitrate as the sole nitrogen source; for samples with 300 mM chlorate, 25 mM ammonium nitrate was used as a nitrogen source. *wt* = wild-type strain 74-OR23-1 V. *Δ nit-9* = *nit-9* knock-out strain.

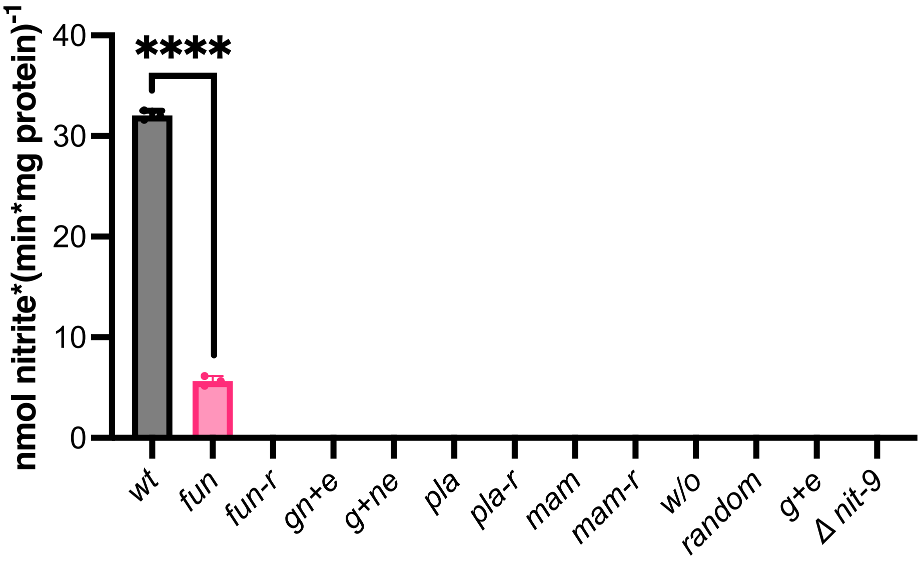

Supplementary Figure 6 | Nitrate reductase activity of strains measured in this study. Nitrate reductase activity in *N. crassa* crude extracts. Strains were grown for 16 h in liquid Vogel's minimal medium (MM) with 80 mM ammonium and subsequently transferred into liquid MM with 80 mM nitrate for 3 h. Experiments were performed in independent triplicates, where each replicate comprised the mean of three technical replicates (n = 3, Mean, Tukey, *P <0.001, One-way ANOVA). N/D = non-detectable activity.

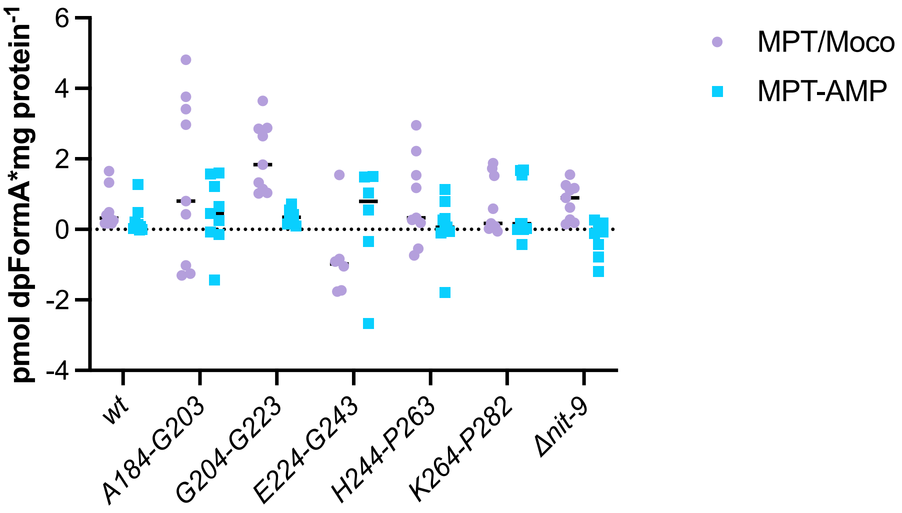

Supplementary Figure 7 | Quantification of MPT-AMP and Moco/MPT content in crude extract from *N. crassa*. Strains were grown for 16 h in liquid Vogel's minimal medium (MM) containing 80 mM ammonium, followed by a 3 h incubation in MM with 80 mM nitrate to induce nitrate assimilation. MPT/Moco and MPT-AMP were oxidized to dephospho-FormA (dpFormA) in an acidic iodine environment, followed by treatment with alkaline phosphatase and phosphodiesterase (MPT-AMP). The dpFormA content, measured via HPLC, is shown as pmol per mg of total protein for the truncation strains, *wt*, and the *Δ nit-9* mutant. For MPT-AMP values, MPT/Moco was previously subtracted from the resulting MPT-AMP signal.

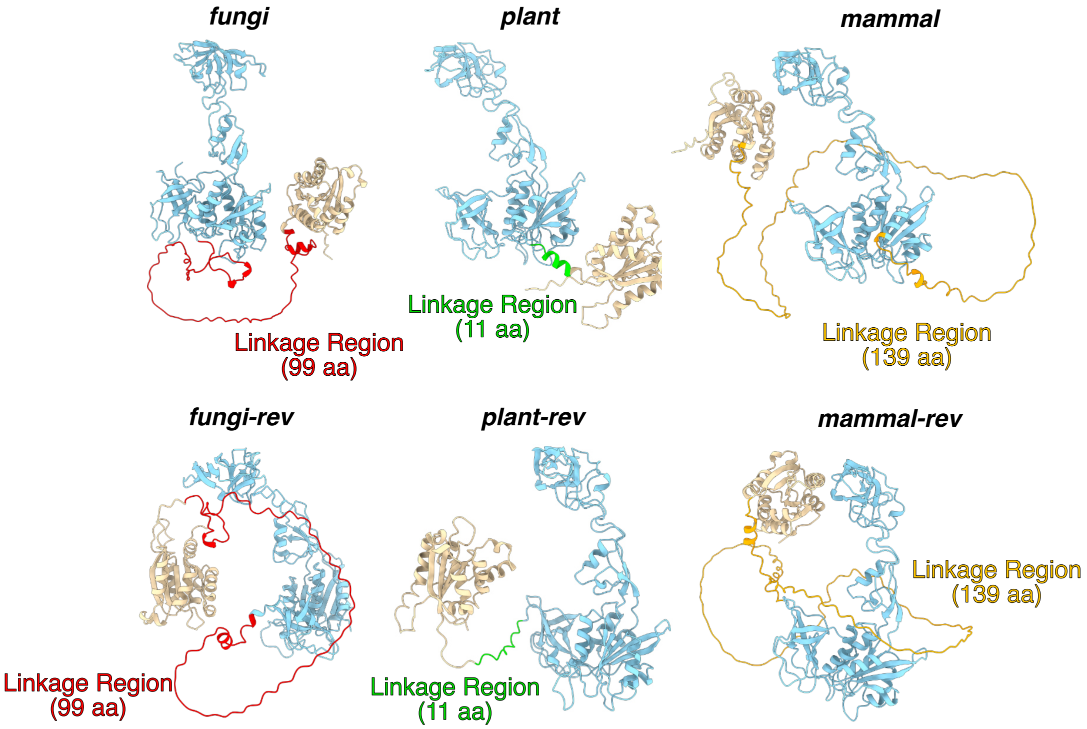

Supplementary Figure 8 | Mo insertase linkage region results in unstructured sequence. AlphaFold 3 models of NIT-9 E and G domain linked with the convergently developed linkage sequences in both orientations as used in growth analysis. The NIT-9E domain is shown in sky blue, the NIT-9G domain in beige, the fungal linkage region from *N. crassa* in red, the plant linkage region from *A. thaliana* in green, and the mammalian linkage region from *H. sapiens* in orange.

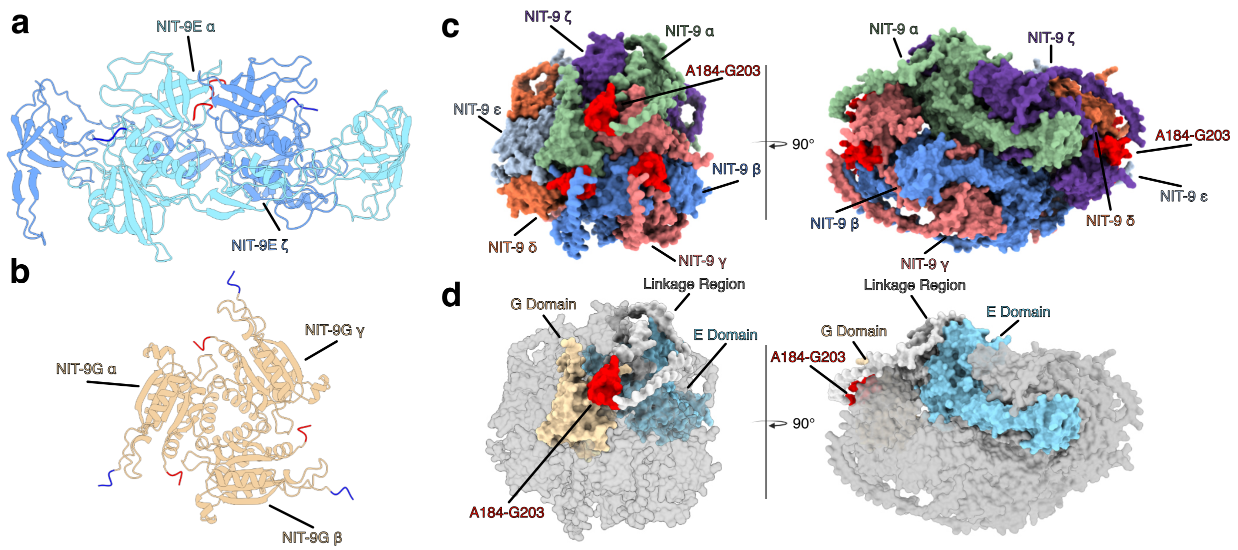

Supplementary Figure 9 | NIT-9 hexameric complex structure is based on G domain trimer and E domain dimer. a, b NIT-9E domain dimer and NIT-9G trimer modeled with AlphaFold 3 superimposed to CNX1E (PDB: 6Q32) and CNX1G (PDB: 1UUX), respectively. The N-terminus is highlighted in blue, and the C-terminus in red. **c,d** AlphaFold 3 models of NIT-9 hexamer. **c** NIT-9 protein complex colored according to protein chains. NIT-9 α green, NIT-9 β blue, NIT-9 γ salmon, NIT-9 δ orange, NIT-9 ε light blue, NIT-9 ζ purple, and A184-G203 in red. **d** One NIT-9 chain is shown in color within the complex, colored according to protein domain. NIT-9E sky blue, NIT-9G beige, the unstructured linkage region in gray, and A184-G203 in red. One NIT-9 chain is shown in color within the complex.

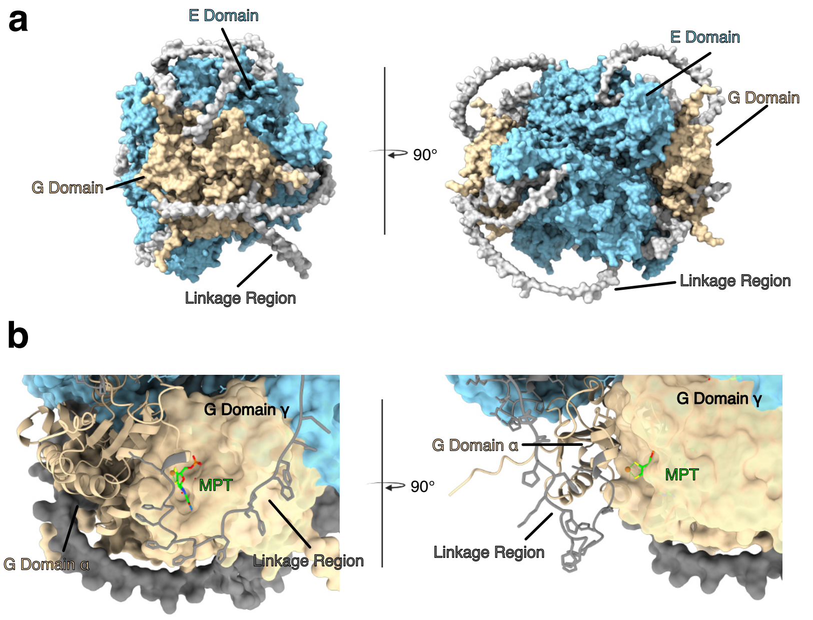

Supplementary Figure 10 | NIT-9 hexamer modelled without A184-G203. a AlphaFold 3 generated protein structure of native NIT-9 hexamer without residues A184-G203. The E domain is depicted in sky blue, the G domain in beige, and the unstructured linkage region in gray. **b** Interface of two G domains in proximity with the linkage region. G domain γ was superimposed (PDB: 1UUX) to reveal the binding position of MPT.

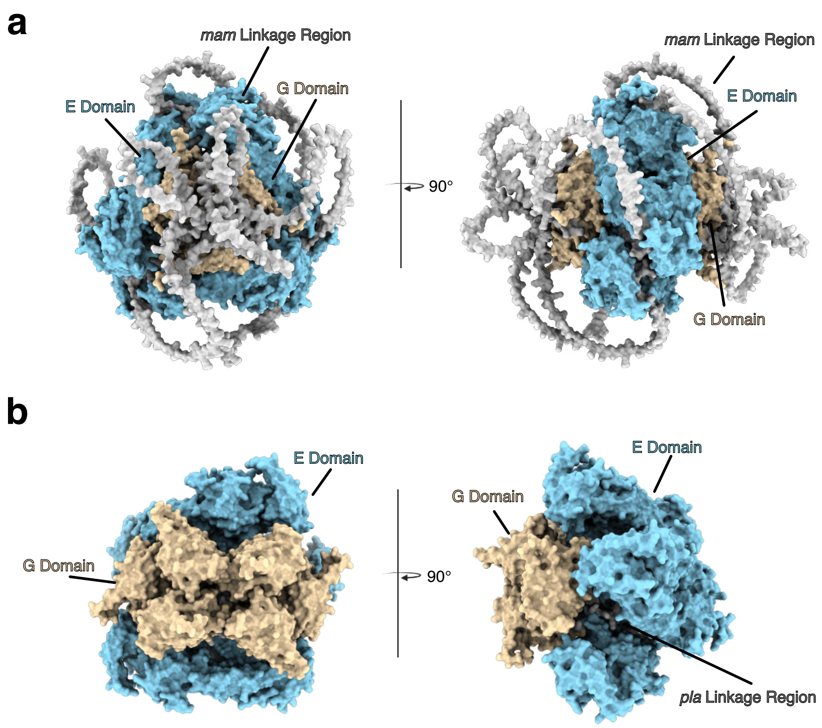

Supplementary Figure 11 | Structural model of NIT-9 hexamers with mammal and plant linkage regions. AlphaFold 3 generated protein structure of NIT-9 variants with convergently evolved linkage sequences, forming hexamers. The E domain is depicted in sky blue, the G domain in beige, and the unstructured linkage region in gray. **a** mammal linkage sequence. **b** plant linkage sequence.

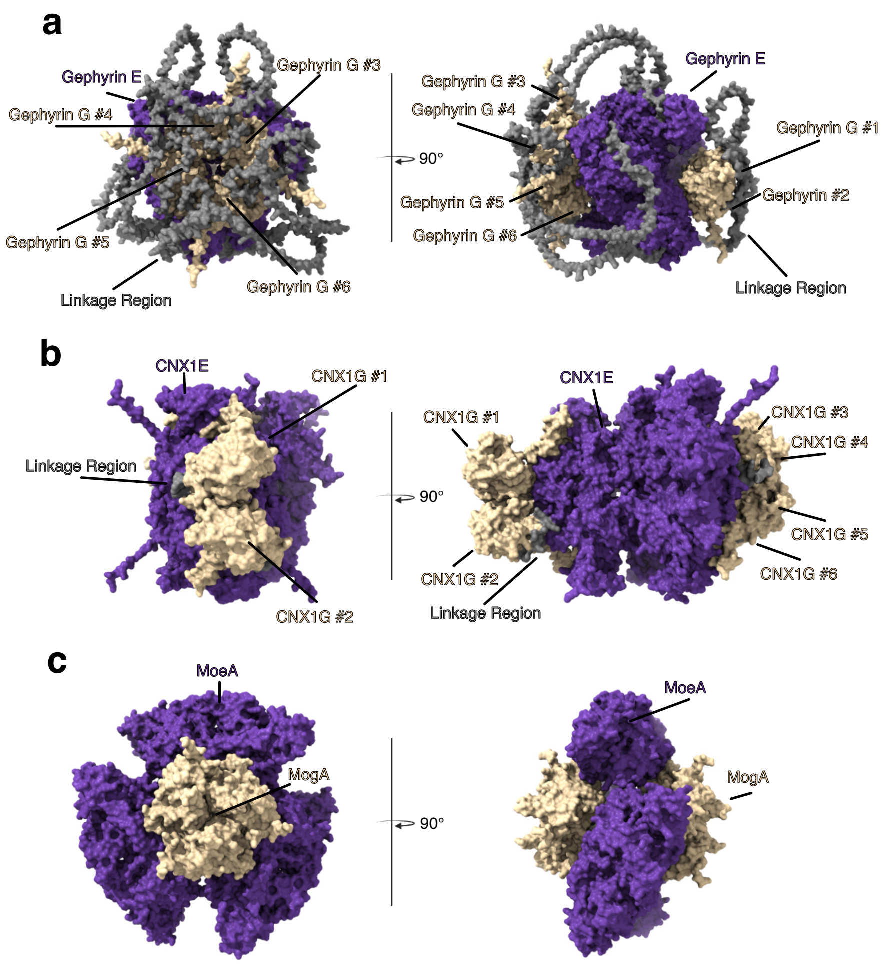

Supplementary Figure 12 | Structural model of Mo insertase hexamers from mammals, plants, and bacteria. AlphaFold 3 generated protein structures of Mo insertase hexamers. The E domain is depicted in purple, the G domain in beige, and the unstructured linkage region in gray. **a** gephyrin from *H. sapiens* (AAF81785.1). **b** CNX1 from *A. thaliana* (NP_197599.1). **c** MogA and MoeA from *E. coli* (NP_414550.1, CAD6018316.1).

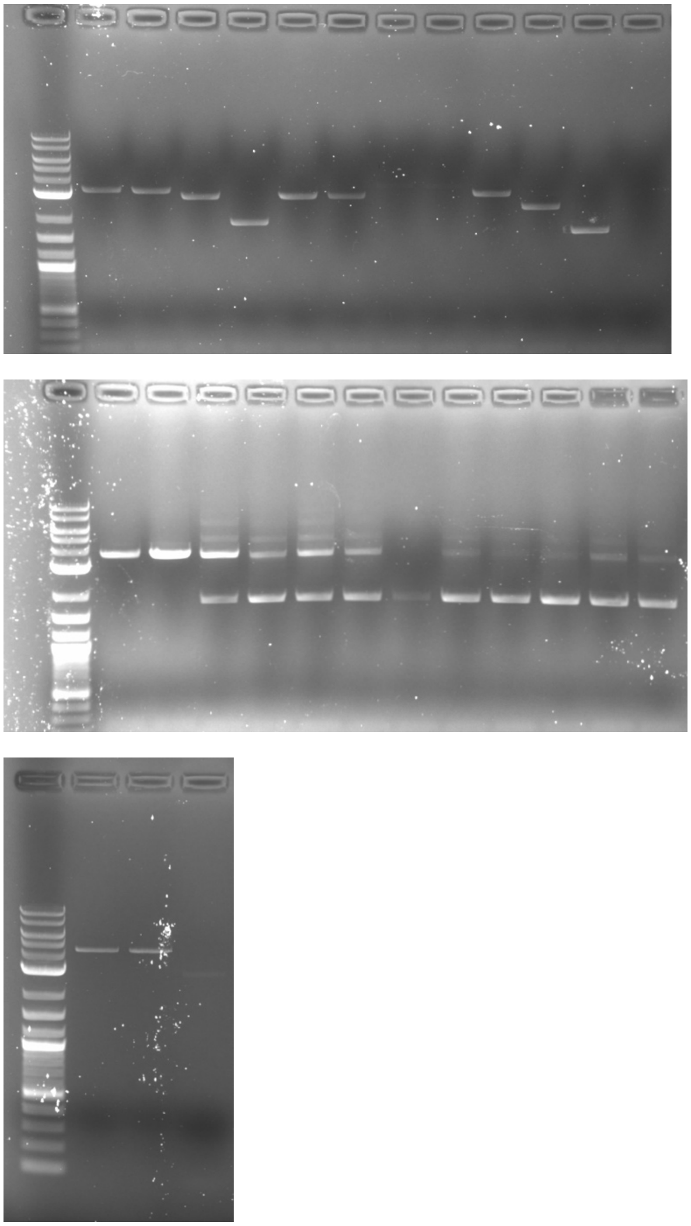

Supplementary Figure 13 | Uncropped Agarose gels of NIT-9 constructs. Gel electrophoresis (1% agarose) was used to confirm transformants by PCR and verify the target gene. Subsequently, the bands were excised and sequenced via Sanger sequencing. The NEB 1kb + ladder (Cat. #N3200S New England Biolabs Ipswitch, USA) was used as reference.

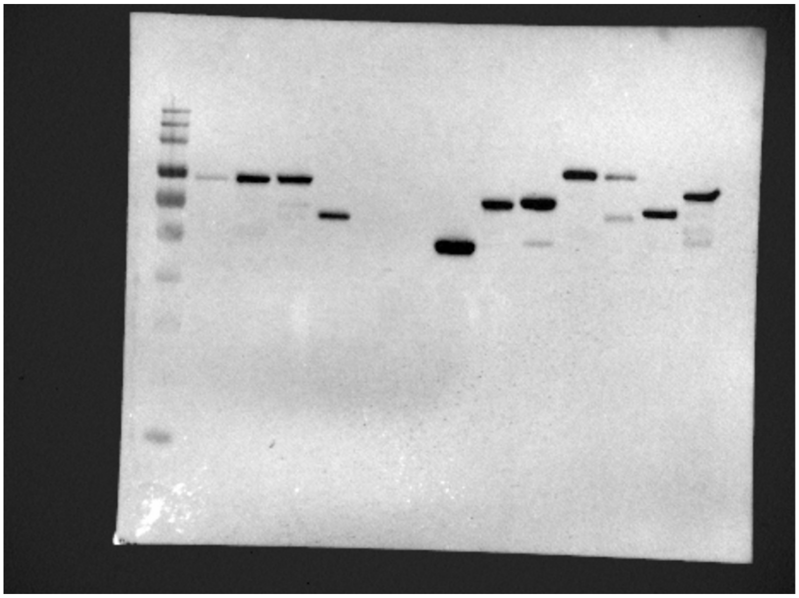

Supplementary Figure 14 | Uncropped Western Blot of NIT-9 constructs. Western Blot to confirm protein expression of the expressed constructs. Each lane was loaded with 50 µg of *N. crassa* crude extract. The wild-type (*wt*) and *nit-9 N. crassa* strains are shown. Detection of the NIT-9E domain was achieved using a specific α-NIT-9E antibody. Size was determined using PageRuler Plus Prestained (Cat. #26620 Thermo Scientific Waltham, USA).

Supplementary Tables

Supplementary Table 1 | Strains used in this study.

Strain ID	Other ID	Mat	Genotype	Construction strategy	Reference (Source)
wt	#2489	mat A	74-OR23-1 VA	-	FGSC
Δ nit-9	#18574	mat a	*nit-9::hph; mus52::bar*	-	FGSC
his-3-	#988	mat A	*his-3-; mus52::bar*	-	FGSC
Δ nit-9; his-3-	K1-57	mat A	*his-3-; mus52::bar, nit-9::hph; mus 52::bar*	crossing	this study
fun	K1-67	mat A	*his-3+::pccg-1::nit-9; nit-9::hph; mus52::bar*	Trafo, integration *his-3*	this study
fun-r	K1-68	mat A	*his-3+::pccg-1::nit-9E::nit-9C::nit-9G; nit-9::hph; mus52::bar*	Trafo, integration *his-3*	this study
pla	K1-72	mat A	*his-3+::pccg-1::nit-9G::cnx1-linker::nit-9E; nit-9::hph; mus52::bar*	Trafo, integration *his-3*	this study
pla-r	K1-73	mat A	*his-3+::pccg-1::nit-9E::cnx1-linker::nit-9G; nit-9::hph; mus52::bar*	Trafo, integration *his-3*	this study
mam	K1-74	mat A	*his-3+::pccg-1::nit-9G::gephyrin-linker::nit-9E; nit-9::hph; mus52::bar*	Trafo, integration *his-3*	this study
mam-r	K1-75	mat A	*his-3+::pccg-1::nit-9E::gephyrin-linker::nit-9G; nit-9::hph; mus52::bar*	Trafo, integration *his-3*	this study
w/o	K1-69	mat A	*his-3+::pccg-1::nit-9G::nit-9E; nit-9::hph; mus52::bar*	Trafo, integration *his-3*	this study
G domain	K1-77	mat A	*his-3+::pccg-1::nit-9G; nit-9::hph; mus52::bar*	Trafo, integration *his-3*	this study
E domain	K1-78	mat A	*his-3+::pccg-1::nit-9E; nit-9::hph; mus52::bar*	Trafo, integration *his-3*	this study
G-linker	K1-71	mat A	*his-3+::pccg-1::nit-9G::nit-9C; nit-9::hph; mus52::bar*	Trafo, integration *his-3*	this study
linker-E	K1-70	mat A	*his-3+::pccg-1::nit-9C::nit-9E; nit-9::hph; mus52::bar*	Trafo, integration *his-3*	this study
A184-G203	K1-79	mat A	*his-3+::pccg-1::nit-9ΔA184-G203 ; nit-9::hph; mus52::bar*	Trafo, integration *his-3*	this study
G204-G223	K1-80	mat A	*his-3+::pccg-1::nit-9ΔG204-G223 ; nit-9::hph; mus52::bar*	Trafo, integration *his-3*	this study
E224-G243	K1-81	mat A	*his-3+::pccg-1::nit-9ΔE224-G243 ; nit-9::hph; mus52::bar*	Trafo, integration *his-3*	this study
H244-P263	K1-82	mat A	*his-3+::pccg-1::nit-9ΔH244-P263 ; nit-9::hph; mus52::bar*	Trafo, integration *his-3*	this study
K264-P282	K1-83	mat A	*his-3+::pccg-1::nit-9ΔK264-P282 ; nit-9::hph; mus52::bar*	Trafo, integration *his-3*	this study
random	K1-76	mat A	*his-3+::pccg-1::nit-9G::mocos-linker::nit-9E; nit-9::hph; mus52::bar*	Trafo, integration *his-3*	this study
gephyrin	K1-84	mat A	*his-3+::pccg-1::gephyrin; nit-9::hph; mus52::bar*	Trafo, integration *his-3*	this study

Supplementary Table 2 | Primers and plasmids used in this study.

Construct	Name	DNA #	Primer Name	Primer Sequence	Parental Vector	Cloning Method
1	*fun*	D128	F_NIT-9.FOR	GGTTAATTAACatgtcctcaagtactactccagcag	PMF272	NEBuilder
			F_NIT-9.REV	TCGAATTCTTAaatcacatcgctgcgtaaacc		
2	*fun-r*	D129	F1_pCCG_NIT-9fl_reverse.FOR	GGTTAATTAACatgctctctgtctccgaagc	PMF272	NEBuilder 4 fragments
			F1_pCCG_NIT-9fl_reverse.REV	ttatgtaaagcaatcacatcgctgcgtaaacc		
			F2_pCCG_NIT-9fl_reverse.FOR	cgatgtgattgctttacataaggggggagtgaagaaa		
			F2_pCCG_NIT-9fl_reverse.REV	tgaggacatcgggtaaggagactcgc		
			F3_pCCG_NIT-9fl_reverse.REV	TATCGAATTCTTAtctagagttagcaccagcagcc		
			F3_pCCG_NIT-9fl_reverse.FOR	cttacccgatgtcctcaagtactactccagcag		
			V_pCCG_NIT-9fl_reverse.FOR	gctaactctagaTAAGAATTCGATATCAAGCTTATCGATACCGT		
			V_pCCG_NIT-9fl_reverse.REV	acagagagcatGTTAATTAACCCGGGGATCCACT		
3	*w/o*	D130	pCCG_NIT-9_GE.FOR	ATGCTCTCTGTCTCCGAAG	D128	Site-directed mutagenesis
			pCCG_NIT-9_GE.REV	TCTAGAGTTAGCACCAGC		
4	*linker-E*	D131	pCCG_Linker_NIT-9E.FOR	GCTTTACATAAGGGGGGAG	D128	Site-directed mutagenesis
			pCCG_Linker_NIT-9E.REV	CATGTTAATTAACCCGGG		
5	*G-linker*	D132	pCCG_NIT-9G-Linker.FOR	TAAGAATTCGATATCAAGCTTATCGATACC	D128	Site-directed mutagenesis
			pCCG_NIT-9G-Linker.REV	CGGGTAAGGAGACTCGCG		
6	*pla*	D133	pCCG_Ni9fl_CNX1.FOR	gtacgatgaagtacctATGCTCTCTGTCTCCGAAG	D128	Site-directed mutagenesis
			pCCG_NIT-9fl_CNX1.REV	ttcttttctttccgaatTCTAGAGTTAGCACCAGC		
7	*pla-r*	D134	R_pCCG_NIT-9fl_CNX1.FOR	GTACGATGAAGTACCTATGTCCTCAAGTACTACTCC	D128	Site-directed mutagenesis
			R_pCCG_NIT-9fl_CNX1.REV	TTCTTTTCTTTCCGAATAATCACATCGCTGCGTAAAC		
8	*mam*	D135	F_pCCG_NIT-9fl_Gephyrin-Linker.FOR	ctaactctagagaacttgaagatttgccttccccac	D128	NEBuilder
			F_pCCG_NIT-9fl_Gephyrin-Linker.REV	cagagagcattcttctagccaccttggtgatatcg		
			V_pCCG_NIT-9fl_Gephyrin-Linker.FOR	gctagaagaatgctctctgtctccgaagc		
			V_pCCG_NIT-9fl_Gephyrin-Linker.REV	atcttcaagttctctagagttagcaccagcagc		
9	*mam-r*	D136	RF_pCCG_NIT-9fl_Gephyrin.FOR	cgatgtgattgaacttgaagatttgccttccccac	PMF272	NEBuilder
			RF_pCCG_NIT-9fl_Gephyrin.REV	ttgaggacattcttctagccaccttggtgatatcg		
			RV_pCCG_NIT-9fl_Gephyrin.FOR	gctagaagaatgtcctcaagtactactccagcag		
			RV_pCCG_NIT-9fl_Gephyrin.REV	cttcaagttcaatcacatcgctgcgtaaacc		
10	*random*	D137	F_pCCG_NIT-9fl_Random.FOR	actctagagactggcctgtccctcagg	PMF272	NEBuilder
			F_pCCG_NIT-9fl_Random.REV	agagagcatcaggactcctgcagctttctct		
			V_pCCG_NIT-9fl_Random.FOR	gagtcctgatgctctctgtctccgaagc		
			V_pCCG_NIT-9fl_Random.REV	aggccagtctctagagttagcaccagcagc		
11	*E domain*	D156	F_pCCG_C-Gly_3xFLAG_NIT-9 E.FOR	TCAACCAAAatgctctctgtctccgaagc	PMF272	NEBuilder
			F_pCCG_C-Gly_3xFLAG_NIT-9 E.REV	CCTCCGCCctaaatcacatcgctgcgtaaaccg		

			V_pCCG_C-Gly_3xFLAG_NIT-9E.FOR	tgatttagGGCGGAGGCGGCGGA		
			V_pCCG_C-Gly_3xFLAG_NIT-9E.REV	cagagagcaF24:K24GATGTGAGGGGTTGTG		
12	*G domain*	D157	pCCG_NIT-9E/G.FOR	TGATCCCGGGTGGCATCC	D128	Site-directed mutagenesis
			pCCG_NIT-9G.REV	TCTAGAGTTAGCACCAGCAGC		
13	*A184-G203*	D164	A184-G203.FOR	GGACGTGGACATGGTCAC	D128	Site-directed mutagenesis
			A184-G203.REV	TCTAGAGTTAGCACCAGC		
14	*G204-G223*	D166	G204-G223.FOR	GAGCACCGAGGAGGAGGA	D128	Site-directed mutagenesis
			G204-G223.REV	ACCACCTCCTTCTACTCCC		
15	*E224-G243*	D167	E224-G243.FOR	CACGGCCCAGGCCACGGC	D128	Site-directed mutagenesis
			E224-G243.REV	GCCATGGTCGTGATGCTTGTGTCCATG		
16	*H244-P263*	D168	H244-P263.FOR	AAGTCCAACGATCCCAGC	D128	Site-directed mutagenesis
			H244-P263.REV	ACCGTGGTTATGGCCATG		
17	*K264-P282*	D169	K264-P282.FOR	ATGCTCTCTGTCTCCGAAG	D128	Site-directed mutagenesis
			K264-P282.REV	GGGGTTTTCAGAGGGGAT		
18	*Gephyrin*	D177	F_GEPH2.FOR	TTAATTAACATGGCGACCGAGGG	PMF272	NEBuilder
			F_GEPH2.REV	ATCGAATTCTCATAGCCGTCCAATGACCATGAC		
			V_GEPH2.FOR	CGGCTATGAGAATTCGATATCAAGCTTATCGATACCG		
			V_GEPH2.REV	CGGTCGCCATGTTAATTAACCCGGGGATCCACT		

Supplementary Table 3 | Accession numbers for Mo insertase genes.

Organism name	Accession number		Mo Insertase name	
	**G domain**	**E domain**	**G domain**	**E domain**
Arabidopsis thaliana	NP_197599.1		CNX1	
Aspergillus nidulans	XP_661382.1		CNXE	
Caenorhabditis elegans	W01A11.6.1	T06H11.4.1	MOC-2	MOC-1
Chlamydomonas reinhardtii	ABC42491.1	ABC42492.1	Cnx1G	Cnx1E
Drosophila melanogaster	NP_477030.1		Cinnamon	
Escherichia coli	NP_414550.1	CAD6018316.1	MogA	MoeA
Homo sapiens	AAF81785.1		Gephyrin	
Methanocaldococcus jannaschii	WP_010869662.1	WP_010870171.1	MoaB	MoeA
Neurospora crassa	XP_011394325.1		NIT-9	
Trichinella spiralis	XP_003379380.1		Gephyrin	
