# Supplementary material for: Convergent evolution links molybdenum insertase domains with organism-specific sequences
Source: Commun Biol. 2024 Oct 18;7:1352. doi: 10.1038/s42003-024-07073-w (PMC11489736; doi:10.1038/s42003-024-07073-w)
Supplement: Supplementary file 2 — Description of Additional Supplementary Materials [file 42003_2024_7073_MOESM2_ESM.pdf]

Description of Additional Supplementary Files

File name: Supplementary Data 1

Description: All numerical source data
